# Genome-wide association studies for growth traits in broilers

**DOI:** 10.1186/s12863-021-01017-7

**Published:** 2022-01-03

**Authors:** Dachang Dou, Linyong Shen, Jiamei Zhou, Zhiping Cao, Peng Luan, Yumao Li, Fan Xiao, Huaishun Guo, Hui Li, Hui Zhang

**Affiliations:** 1 Key Laboratory of Chicken Genetics and Breeding, Ministry of Agriculture and Rural Affairs; Key Laboratory of Animal Genetics, Breeding and Reproduction, Education Department of Heilongjiang Province; College of Animal Science and Technology, Northeast Agricultural University, Harbin, 150030 P. R. China; 2 Fujian Sunnzer Biotechnology Development Co., Ltd, Nanping, 354100 P. R. China

**Keywords:** Broiler, Growth trait, Body weight, GWAS, Heritability

## Abstract

**Background:**

The identification of markers and genes for growth traits may not only benefit for marker assist selection /genomic selection but also provide important information for understanding the genetic foundation of growth traits in broilers.

**Results:**

In the current study, we estimated the genetic parameters of eight growth traits in broilers and carried out the genome-wide association studies for these growth traits. A total of 113 QTNs discovered by multiple methods together, and some genes, including *ACTA1*, *IGF2BP1*, *TAPT1*, *LDB2*, *PRKCA*, *TGFBR2*, *GLI3*, *SLC16A7, INHBA, BAMBI, APCDD1, GPR39,* and *GATA4*, were identified as important candidate genes for rapid growth in broilers.

**Conclusions:**

The results of this study will provide important information for understanding the genetic foundation of growth traits in broilers.

**Supplementary Information:**

The online version contains supplementary material available at 10.1186/s12863-021-01017-7.

## Background

The chicken (*Gallus gallus*), as an important model animal, could fulfill the evolutionary gap among mammals and other vertebrates. Some substantial advances have been made to improve the growth rate in chicken for the past more than 60 years under artificial selection and rapid growth in broilers will continue to be the most important economic trait in breeding programs. Growth traits in chicken often have high heritability and phenotypic selection may obtain good selection progress. However, the identification of markers and genes for growth traits may not only benefit for marker assist selection (MAS)/genomic selection (GS) but also provide important information for understanding the genetic basis of growth traits in broilers.

Genome-wide association studies (GWAS) is a technology to identify loci significantly associated with traits of interested by using single nucleotide polymorphisms (SNP) chips or sequencing technology which screen hundreds of thousands or even millions of SNPs or some other kinds of variants in the genomes. This approach is first used in human to detect causal mutations for disease and until now many loci for diseases, especially for many kinds of cancers, are identified [1, 2]. GWAS is also implemented in domestic animals to identify the genetic factors associated with economically important traits [3–5]. Thanks to the advantage of SNP arrays and genomic resequencing methods for animals, it is relatively easy to genotype a wide array of individuals. As a result, many GWAS have been carried out in the past few years. In chickens, a number of markers or genes important for growth, meat quality, and fertility traits, were identified in different populations by using GWAS [6–9].

Our team constructed the Northeast Agricultural University broiler lines divergently selected for abdominal fat content (NEAUHLF) since 1996 [10]. Plasma VLDL concentration of individual and its fullsibs’ abdominal fat percentage (AFP) were used as criteria. Using this population, we carried out the GWAS analysis for plasma VLDL concentration and for fertility traits [11, 12]. For growth rate, an important quantitative trait in broilers, it is important to identify markers or genes that could be used in breeding program to improve it quickly. Therefore, in the current study, GWAS was used to identify important genes for growth traits in NEAUHLF. The results of this study will provide important information for understanding the genetic background of growth traits in broilers.

## Results

### Genetic parameter estimation of growth traits in broilers

**Fig. 1:**
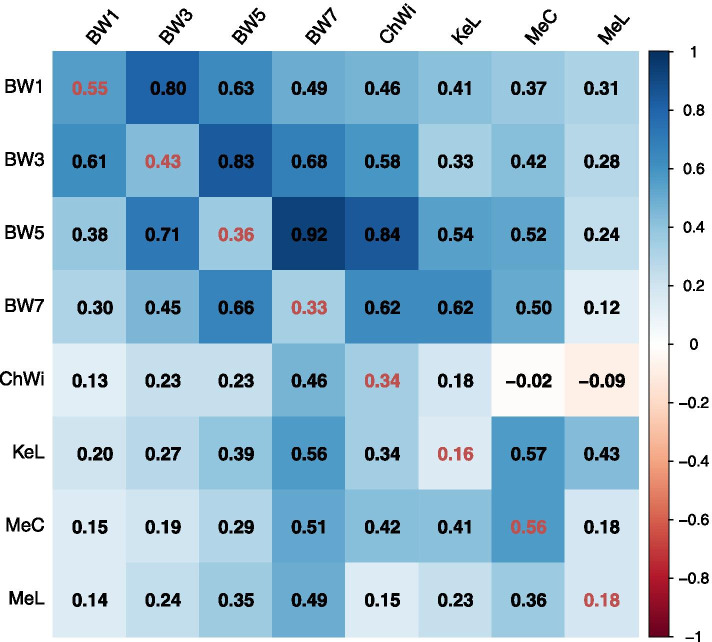
Genetic parameters of the eight growth traits in broilers. Heritability was on the diagonal. Above the diagonal were the genetic correlations between every two traits and below the diagonal were the phenotypic correlations

The descriptive information of the eight growth traits, including BW1, BW3, BW5, BW7, ChWi, KeL, MeC and MeL, was shown in Table 1. The results indicated that BW7 used in the current study was about 2.4 kg. The heritability of these eight growth traits were estimated and we found that six of these traits, including BW1, BW3, BW5, BW7, ChWi and MeC, have high heritability (*h*^*2*^ > 0.3) (Fig. 1). However, KeL (*h*^*2*^ = 0.16) and MeL (*h*^*2*^ = 0.18) have a little low heritability. The genetic and phenotypic correlation between body weight at different weeks of age were high (*r* > 0.3) (Fig. 1). Overall, the genetic correlation between every two of the eight growth traits were moderate or high except several low ones, such as the genetic correlation between ChWi and MeC, and between ChWi and MeL were − 0.02 and − 0.09, respectively (Fig. 1).

**Table 1:** Descriptive information of growth traits in broilers

Growth traits	Mean	Standard deviation	Max	Min	C.V. (%)	Phenotypic variance	Genetic variance
BW1 (g)	122.00	12.33	174.60	70.10	10.11	157.64	86.12
BW3 (g)	615.22	65.90	797.00	332.00	10.71	4527.37	2564.09
BW5 (g)	1491.19	142.38	1815.00	940.00	9.55	20,517.80	7370.30
BW7 (g)	2400.97	221.41	3020.00	1755.00	9.22	49,804.20	16,163.70
ChWi (cm)	9.23	0.74	11.84	7.21	8.02	0.48	0.16
KeL (cm)	13.75	0.73	18.84	9.96	5.31	0.45	0.07
MeC (cm)	5.10	0.39	6.35	4.35	7.65	0.06	0.03
MeL (cm)	9.25	0.46	10.54	7.75	4.97	0.14	0.03

### GWAS results for growth traits in broilers

**Fig. 2:**
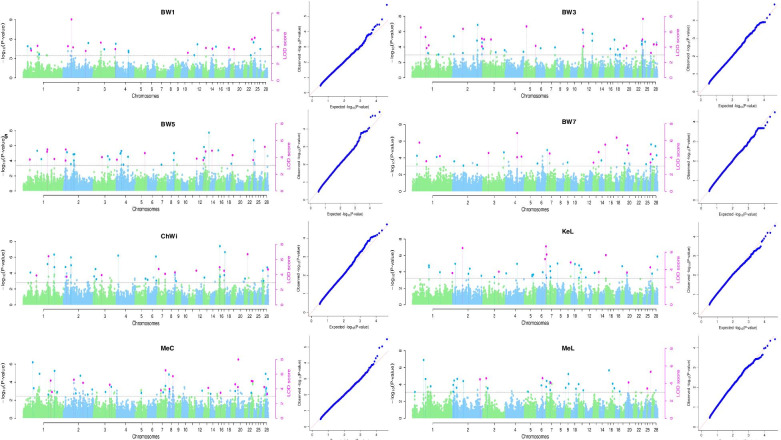
Manhattan and QQ plots for eight growth traits in GWAS using mrMLM v4.0.2. Left is Manhattan plot, while right is QQ plot. Loci discovered by multiple methods together are marked with pink dots in the Manhattan diagram, those discovered by a single method are marked in dark blue, and the horizontal line indicates a critical LOD score of 3.0. BW1, 3, 5, 7 = body weight at 1, 3, 5 and 7 weeks of age; ChWi = chest width; KeL = keel length; MeC = metatarsus circumference; MeL = metatarsus length

The six multi-locus GWAS methods in the mrMLM v4.0.2 package, were used to carry out the GWAS analysis for the eight growth traits of 475 male birds from the 11th generation of NEAUHLF (Fig. 2). We identified 285 quantitative trait nucleotides (QTNs) with significant effects on eight of the growth traits, including BW1, BW3, BW5, BW7, ChWi, KeL, MeC and MeL, based on a logarithm of odds (LOD) threshold of ≥3. Of these QTNs, 113 ones were discovered by multiple methods together (at least two methods) (Table S1). These significant QTNs for growth traits were distributed on chromosomes 1, 2, 3, 4, 6, 7, 8, 12, 13, 15, 19, 20, 23, and 26 (Fig. 2).

### Difference of phenotypes between different alleles

**Fig. 3:**
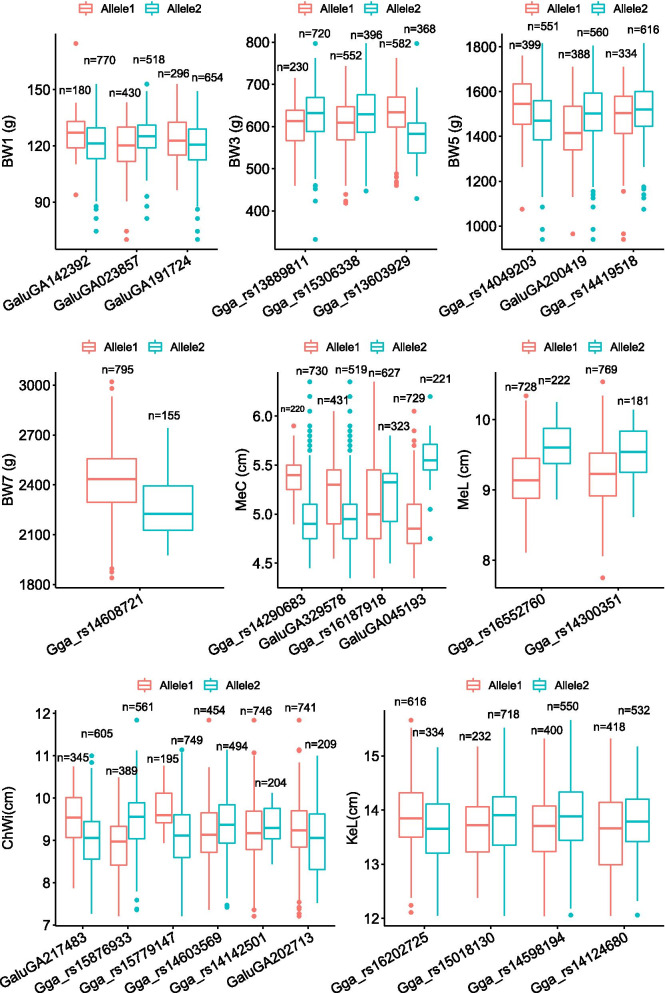
Phenotypic differences of growth traits in broilers between two alleles of significant QTNs

A total of 26 QTNs with significant effects on growth traits were detected by at least four multi-locus GWAS methods, including 3, 3, 3 and 1 significant QTNs for body weight at 1, 3, 5 and 7 weeks of age, respectively, and 6 QTNs for ChWi, 4 QTNs for KeL, 4 QTNs for MeC and 2 QTNs for MeL. The difference of growth traits between two alleles of the QTNs with significant effects were calculated. The results indicated that birds with different alleles have significantly different (*P* < 0.05) phenotypes (BW1, BW3, BW5, BW7, ChWi, KeL, MeC and MeL) for all 26 QTNs (Fig. 3).

### Candidate genes for growth traits in broilers

**Fig. 4:**
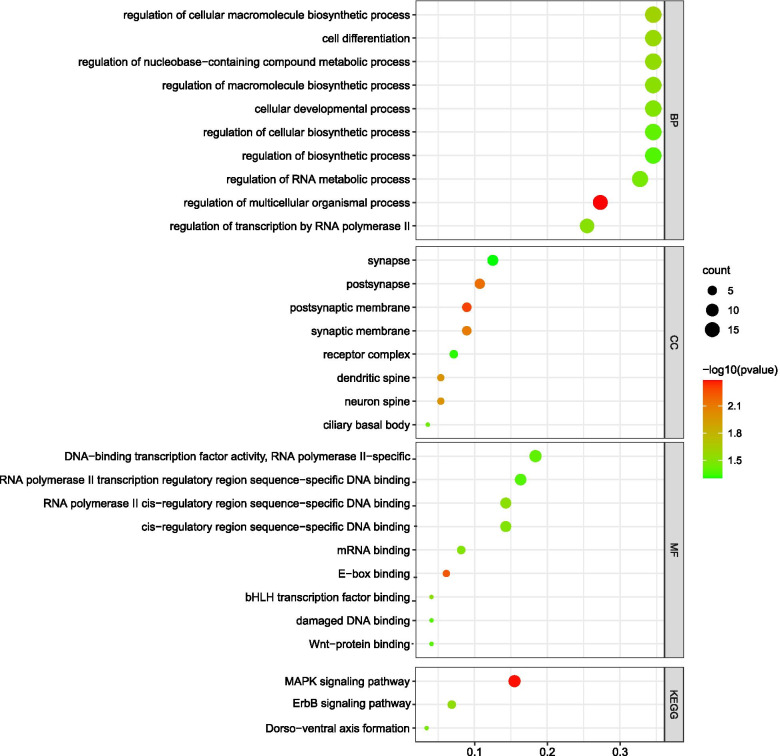
Significant GO categories and KEGG pathways of the genes around QTNs for growth traits in broilers

A total of 184 chicken Refgenes were harbored in 1 Mb regions surrounding the 113 QTNs with significant effects on growth traits by multiple methods together (Table S1). Using these 184 genes, the GO and KEGG analyses were carried out. The results indicated that twenty-seven GO terms reached the statistically significant level (*P* < 0.05) (Fig. 4). These GO terms were mainly involved in cell differentiation, regulation of cellular macromolecule biosynthetic process, postsynaptic membrane, RNA polymerase II-specific and *etc.* Only three KEGG pathways reached the significant level according to the KEGG pathway analyses (Fig. 4). The papers about these 184 genes were found and some genes, including Acetyl Coenzyme A Acetyltransferase 1 (*ACTA1*), Insulin-Like Growth Factor 2 MRNA Binding Protein 1 (*IGF2BP1*), Transmembrane Anterior-Posterior Transformation 1 (*TAPT1*), LIM Domain Binding 2 (*LDB2*)*,* Protein Kinase C Alpha (*PRKCA*), Transforming Growth Factor Beta Receptor 2 (*TGFBR2*), GLI Family Zinc Finger 3 (*GLI3*), Solute Carrier Family 16 Member 7 (*SLC16A7*), Inhibin Subunit Beta A (*INHBA*)*,* BMP And Activin Membrane-Bound Inhibitor (*BAMBI*), (APC Down-Regulated 1 *APCDD1*)*,* G Protein-Coupled Receptor 39 (*GPR39*) and GATA Binding Protein 4 (*GATA4*), were selected as candidates for broiler growth based on these results.

## Discussion

Growth trait, especially body weight, is the most important economic trait in the poultry industry. In the current study, we measured eight growth traits in broilers and the genetic parameters of these traits were estimated. We found that the heritability of body weight at different weeks of age was high (*h*^*2*^ > 0.3), which is consistent with previous studies, such as Venturini et a1 [13]. found that the heritability of BW5 and BW6 were 0.41 and 0.45 respectively; Kapell et al. [14, 15] estimated the heritability of body weight which ranged from 0.326 to 0.399; Mebratie et al. [16] found that the heritability of body weight ranged from 0.31 to 0.37 at different weeks of age in broiler chickens; and Chu et al. [17] found that the heritability of body weight of broilers ranged from 0.28 to 0.33 at different weeks of age. However, the results of some other studies, which found that whereas in our study they ranged from 0.156 to 0.187 [18], were different from the current study. The differences in the results from these studies may be because that the birds used in these studies have different genetic backgrounds and the population sizes were also different. The current study found that the genetic and phenotypic correlations between different growth traits had a large range. Therefore, it is important to pay much more attention when editing the breeding program because when we select one trait the other traits may also have selection responses.

In this study, we used the six multi-locus GWAS methods applied by mrMLM v4.0.2 package to identify SNPs significantly associated with growth traits in broiler chickens. The mixed linear model (MLM) approach has been widely used in GWAS because it can effectively control the false positive rate of SNP detection. Bonferroni correction is a common approach in single-locus GWAS models, but it assumes that markers are independent of each other. Bonferroni correction is too stringent for growth trait data so that many important small effect loci are lost [19, 20]. In order to solve this problem, Zhang et al. [21] developed an R software mrMLM v4.0.2, including six multi-locus methods (mrMLM [19], FASTmrMLM [21], FASTmrEMMA [20], ISIS EM-BLASSO, pLARmEB, and pKWmEB). Since the number and effects of all potentially associated markers can be determined and estimated simultaneously in the six multi-locus methods, no Bonferroni correction is required. Although the LOD score threshold of 3.0 is set, it can control the false positive rate well and obtain high statistical power.

A total of 113 QTNs with significant effects on growth traits by multiple methods (at least two methods) together in the current study and these QTNs were located on chicken chromosomes 1, 2, 3, 4, 6, 7, 8, 12, 13, 15, 19, 20, 23, and 26. GWAS for body weight was also carried out in some other populations in chicken and these results had some overlaps with the current study. Mebratie et al. [22] carried out the GWAS analysis for body weight and found that QTNs with significant effects on body weight were located on chicken chromosomes 1, 6, 8, 12, 14, 23, and Z, which overlapped with the current study. Xu et al. [23] reported that chromosomes 1 and 4 are the two critical chromosomes influencing growth traits particularly body weight in chickens according to the results of GWAS analysis. Van et al. [24] also identified some QTNs with significant effects on body weight located on chromosomes 1, 2, 3, 4, 5, 6, 7, 8, 14, and 26. The phenotype differences between two alleles of the 26 QTNs with significant effects on growth traits by at least four multi-locus GWAS methods were calculated and the results showed that birds with different alleles have significantly different phenotypes (growth traits) for all 26 QTNs. These results indicated that these QTNs could be used in MAS/GS to select rapid growth in broilers.

There were 184 Refgenes of chicken in 1 Mb region of these 113 QTNs with significant effects on growth traits. The basic function of these 184 genes was extracted from the previous reports and some genes, including *ACTA1*, *IGF2BP1, TAPT1, LDB2, PRKCA, TGFBR2*, and *GLI3*, which were reported to be associated with growth traits in farm animals, were selected as important candidate genes for growth traits in broilers. The results of association study showed that *ACTA1* can be used together with others already described to increase the economically important traits in broilers [25]. Three InDels were identified on *IGF2BP1* gene that were significantly associated with growth traits in sheep [26]. The polymorphisms of *TAPT1* were significantly associated with carcass weight and eviscerated weight in broilers [27]. The 31-bp InDel of the *LDB2* gene was significantly correlated with multiple growth and carcass traits in the F_2_ resource population and affected the expression of *LDB2* in muscle tissue [28]. The *PRKCA* gene was associated with intramuscular fat content in bovine muscle tissue [29]. The *TGFBR2* gene plays a negative role in the growth of the scallop, which had a SNP in the 3′ UTR that gives the scallop higher growth performance [30]. The *GLI3* gene was important for the development of the mammalian brain and lungs [31]. Furthermore, some other genes, such as *SLC16A7, INHBA, BAMBI, APCDD1, GPR39, GATA4* and etc., may also be related to growth traits based on their results on humans and mice [32–38].

## Conclusions

In summary, in this study, we estimated the genetic parameters of eight growth traits in broilers and carried out the GWAS analysis for these growth traits. A total of 113 QTNs with significant effects were detected by multiple methods (at least two methods), and some genes, including *ACTA1*, *IGF2BP1*, *TAPT1*, *LDB2*, *PRKCA*, *TGFBR2*, *GLI3*, *SLC16A7, INHBA, BAMBI, APCDD1, GPR39,* and *GATA4* were identified as important candidate genes for rapid growth in broilers.

## Methods

### Sampling

Two Northeast Agricultural University broiler lines divergently selected for abdominal fat content (NEAUHLF) were used in this study. A total of 475 male birds (203 and 272 from the lean and fat lines, respectively) from the 11th generation of NEAUHLF were selected to carry out the genome wide association study (GWAS). The same environmental conditions and free access to feed and water were supplied to all these birds. Commercial corn-soybean-based diets which met the requirements of National Research Council were provided. A starter feed (3000kal ME = kg and 210 g = kg CP) was supplied to birds from hatch to 3 weeks of age and a grower diet (3100 kal ME = kg and 190 g = kg CP) was supplied to birds from 4 weeks of age to slaughter. For reporting of results, we complied with the Animal Research: Reporting In Vivo Experiments guidelines [39]. The birds were weighed at 0 (birth), 1, 3, 5 and 7 weeks of age (BW0, BW1, BW3, BW5 and BW7). At 7 weeks of age, the metatarsus length (MeL), metatarsus circumference (MeC), keel length (KeL) and chest width (ChWi) were measured before slaughter as previously described [40]. Genotypes were obtained using the Illumina chicken 60 K SNP chip which containing a total of 57,636 SNPs. Quality control was carried out with criteria of a call rate ≥ 95% and minor allele frequency (MAF) ≥ 0.05 and a total of 48,824 SNPs were left for GWAS analysis.

### Statistical analysis

The difference of phenotypes between two alleles of every SNPs was calculated using t-test. The genetic parameters, including the heritability of growth traits and the genetic and phenotypic correlations between every two of these growth traits, were estimated using Wombat software [41], with line treated as the fixed effects. Heritability of these traits were estimated using a single-variate model, and the bivariate model was used to calculate the genetic and phenotypic correlations. The genetic model used for parameter estimations is described as follows:

$$\mathrm{y}=\mathrm{X}\upbeta +\mathrm{Zu}+\mathrm{e}$$

in which y is an n-dimensional vector of observed values for the traits, X is an n × p matrix of the fixed effects, β is a p-dimensional vector of the fixed effects, Z is an n × q matrix of the random effects, u is a q-dimensional vector of the random genetic effects, and e is an n-dimensional vector of the random residual effects.

The random effects u and e were assumed to follow the normal distributions with mean 0, that is, Expectation [y] = Xβ. The variances of u and e were assumed to be Var(u) = Ag and Var(e) = Ir, respectively, in which A is the numerator relationship matrix of all animals in the pedigree file, g is the additive genetic variance for the single-variate and the additive genetic variance–covariance matrix between traits for the bivariate model analysis, I is the identity matrix of order equal to the number of animals with phenotypes, and r is the residual variance for the single-variate and the variance–covariance matrix between residuals on the same animal when performing the bivariate model analysis, where residual covariance equal to 0 [42]. The SNP makers were also used to calculate the genetic correlation between every two traits, which means that the individual correlation matrix (A) from pedigree information was replaced by genomic information (G matrix). And the results from G matrix were described in Fig. 1.

The Genome-wide Rapid Association using multi-locus GWAS methods in the mrMLM v4.0.2 package, [21] was used to carry out GWAS. This mixed model contained Line (two broiler lines) and BW0 as covariates. Default values were used for all parameters.

### Gene detection and functional annotation

SNPs with significant effects on growth traits were detected by using GWAS method as above and the genes located in 1 Mb region of these significant SNPs were retrieved from UCSC (https://genome.ucsc.edu/) (Galgal6). The Gene Ontology (GO) terms and Kyoto Encyclopedia of Genes and Genomes (KEGG) pathway analysis were carried out using DAVID bioinformatics resources 6.8 (http://david.abcc.ncifcrf.gov/summary.jsp) for these genes [43–46]. *P*-value <0.05 was used as the statistical significance level.

## Supplementary Information

**Additional file 1: Table S1.** Significant QTNs for eight different growth traits in broilers co-detected at least two multi-locus GWAS methods. ^1^*QTNs in bold font* are pleiotropic QTNs which were detected associate with multiple traits. ^2^Chicken Refgenes in bold font indicates multiple occurrences of the gene.

## Data Availability

The data sets supporting the results of this article are included within the article and its additional files. The chicken 60 k SNP data presented in this paper have been deposited into Gene Expression Omnibus (http://www.ncbi.nlm.nih.gov/geo/) with the identifier GSE58551.

## Abbreviations

MAS: Marker assist selection
GS: Genomic selection
GWAS: Genome-wide association studies
SNP: Single nucleotide polymorphism
QTN: Quantitative trait nucleotide
NEAUHLF: Northeast Agricultural University broiler lines divergently selected for abdominal fat conten
AFP: Abdominal fat percentage
BW: Body weight
MeL: Metatarsus length
MeC: Metatarsus circumference
KeL: Keel length
ChWi: Chest width
MAF: Minor allele frequency
MLM: Mixed linear model
GO: Gene Ontology
KEGG: Kyoto Encyclopedia of Genes and Genomes
